# Measurement of stainer bath contamination and evaluation of common mitigation strategies

**DOI:** 10.1179/2046023612Y.0000000013

**Published:** 2012-09

**Authors:** Angie Cahill, Jeff Pearson

**Affiliations:** Ventana Medical Systems, Inc., Tucson, AZ, USA

**Keywords:** Anatomy, Artifact, Eosin, Equipment contamination, Hematoxylin, Histology

## Abstract

Methods relative to the staining of tissues using hematoxylin and eosin (H&E) have largely not evolved beyond linear batch staining processes. The batching of slides in the histopathology laboratory inherently leads to the sharing of the various reagents among those specimens being processed through the baths. Studies analyzing the effects of reagent sharing during the common H&E linear staining method are limited. This study assessed rates of extraneous tissue contamination found in selected stainer bath containers from the deparaffinization portion of the H&E linear staining procedure. The impact of common mitigation strategies on those rates of contamination was evaluated.

## Introduction

**Figure 1:**
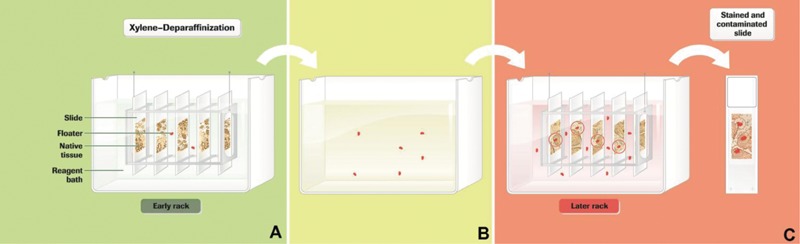
Tissue cross-contamination in linear hematoxylin and eosin (H&E) batch staining. (A) Loaded slide staining racks travel through a given H&E staining bath. (B) Tissue fragments from slides shown in (A) dislodge from the slide(s) and float in the reagent. (C) As successive racks are introduced into the bath, those fragments can adhere to another patient’s slide.

The commonly used ‘linear’ staining practice is either manual or automated. Most laboratories use some level of automation for batch staining, usually an automated instrument that transfers the slide racks from one stainer bath container to the next. This sequential hematoxylin and eosin (H&E) batch staining method can produce quality control issues in the stainer bath containers, most notably tissue cross-contamination and reagent carryover from container to container (Fig. 1). The standard practices for minimizing tissue cross-contamination have been changing reagents or filtering them. Reagent turnover in this study means either changing or filtering reagents.

Few studies exist regarding tissue cross-contamination specific to linear staining. Investigations to date have looked broadly at the sources of extraneous tissue contamination in the histology laboratory, including contamination during gross tissue examination, and general strategies for mitigating contamination during tissue preparation.^1^^–^3 A study published in 2009 by Hunt and coworkers,^1^ specifically examined the commonly held belief that contamination not present in the paraffin block must be generated from the waterbath during microtomy. Their study demonstrated a much greater presence of tissue cross-contamination in the linear stainer bath containers when compared to the waterbath.

Extraneous tissue contamination has been described in publications by Dimenstein,^2^ Gephardt and Zarbo,^4^ and Carpenter.^5^ As noted by Carpenter and Dimenstein, many laboratory technologists and pathologists considered extraneous tissue fragments more of a nuisance than a diagnostic challenge. These extraneous tissue fragments or cellular material, also known as ‘floaters’, are often recognized and dismissed by the pathologist due to the obvious differences in tissue type. As part of a 1996 College of American Pathologists (CAP) Q-Probes study, Gephardt and coworkers confirmed through prospective and retrospective studies the presence of extraneous tissue contaminants on patient slides. The retrospective portion of their study included a population of 57,083 slides and considered the degree of extraneous tissue contamination and diagnostic difficulty. Extraneous tissue was present on 1,653 slides and severe diagnostic difficulty was demonstrated in 0..1% of the slides.^4^ Gephardt found that extraneous tissue contamination was indeed a defect in the linear slide staining process regardless of the outcome for the patient.

## Materials and Methods

### Study protocol

Participants in the study included 69 histopathology laboratories in six different countries within North America, Asia Pacific, and Europe. Two participating laboratories submitted contents from multiple stainers, therefore a total of 72 stainers were studied. Stainer bath contents were collected from laboratories between February 2009 and December 2010. Selection of the reagent baths for examination and analysis and the use of blank slides to quantify cross-contamination in the stainer baths were based on previous methods described by Hunt and coworkers^1^ Each collection of stainer bath contents was supervised by a trained study representative. Both clinical hospital and reference laboratories were represented with annual H&E slide production volumes ranging from 20 000 to 2 184 000. Commercially available linear stainer brands were used in the study. One study participant stained slides using a manual method.

Hunt and coworkers found that the deparaffinization portion of linear bath staining had the greatest potential for extraneous tissue contamination. A study of selected stainer bath contents was performed, based on Hunt’s findings, from deparaffinization solutions used in H&E staining of routine tissue samples. Participating laboratories were asked to disclose the reagent turnover frequency in their linear stainers.

Step 1: Processing blank patient simulator slides with H&E staining run

1. Each laboratory interspersed blank, charged patient simulator slides (VWR^®^ Superfrost^®^ Plus slides; VWR, Radnor, PA, USA) with the last routine H&E batched patient slides of the day.
2. The patient simulator slides were stepped through the laboratory’s linear H&E staining protocol, coverslipped, and mounted with permanent mounting medium using either an automated coverslipper or a manual method.

Step 2: Collection of selected stainer bath contents

1. The contents of the first reagent bath, up to 500 ml, were collected. The variability in stainer bath size and capacity, because of brand and model differences, affected the collection of the deparaffinization solutions. If there was less than 500 ml in the first bath, the contents of the second clearing agent reagent bath were also collected, for a total sample volume of 500 ml.
2. The contents of the 100% alcohol and 95% alcohol baths (maximum 500 ml each) were collected in the same manner.

Step 3: Processing selected stainer bath contents and preparing CytoSpin slides

1. The collected reagents were transported to a centrally located laboratory for in-house preparation and analysis. The homogenized solutions were divided equally into 225 ml centrifuge tubes, and then centrifuged and reduced to a supernatant and pellet (Beckman Coulter Allegra^®^ 6 Centrifuge; Beckman Coulter, Inc., Brea, CA, USA).
2. The supernatant was discarded, and the resulting pellet was resuspended in 5 ml of 100% reagent-grade alcohol. CytoSpin slides were then prepared by dispensing 130 μl of the resuspended pellet from a centrifuge tube into each well of a cytofunnel (Cytology Funnels; VWR) mounted on an uncharged glass microscope slide (Shandon® Double CytoSpin^®^ slides; Thermo Fisher Scientific, Erie Scientific, Portsmouth, NH, USA). The cytofunnel, its contents, and the slide were centrifuged at 500 rev/minute for 5 minutes (Thermo Shandon CytoSpin® 4 Cytocentrifuge; Thermo Fisher Scientific, Erie Scientific). The slides were then dried overnight at room temperature and stained with an automated slide staining system using a progressive staining method.

Step 4: Screening and evaluation of patient simulator slides and CytoSpin slides

1. The blank patient simulator slides from Step 1 of the study protocol were pre-screened by a qualified technologist. Any slides containing extraneous tissue fragments were confirmed by an outside board-certified, third-party pathologist.
2. The CytoSpin slides created from the reagent bath contents in Step 2 were evaluated for fragment count and tissue characterization, e.g. organ type, benign, atypical, or malignant, by the same outside board-certified, third-party pathologist. In qualifying both the patient simulator slides and CytoSpin slides as contaminated, individual keratinocytes (nucleated or anucleated), paraffin remnants, and hematoxylin precipitate were excluded as contaminants.

## Results

A total of 69 laboratories submitted stainer bath contents from 72 stainers that included the clearing agent, 100% alcohol, and 95% alcohol (maximum 500 ml each) and patient simulator slides for evaluation. Results are summarized in Tables 1 and 2. The CytoSpin slides prepared from the stainer bath contents demonstrated the presence of floaters, including tissue fragments and tissue characterization, such as organ type, benign, atypical, or malignant. The number of contaminated patient simulator slides from the study laboratories exhibited tissue migration within the linear stainer baths.

**Table 1:** Summary of contaminated patient simulator slides and stainer bath contents

Total number of contaminated patient simulator slides	164
Total number of qualified floaters in reagent baths	18 145
Average number of tissue types (per linear batch stainer)	8

**Table 2:** Statistical analysis of contaminated patient simulator slides and stainer bath contents

*n* = 72	Mean	Maximum	Minimum	Standard deviation
Percent of contaminated patient simulator slides	2.34	36.4	0	4.88
Number of tissue fragments in reagent baths	250.2	3018	3	490.4
Number of tissue types in reagent baths	7.93	18	2	3.32

**Figure 2:**
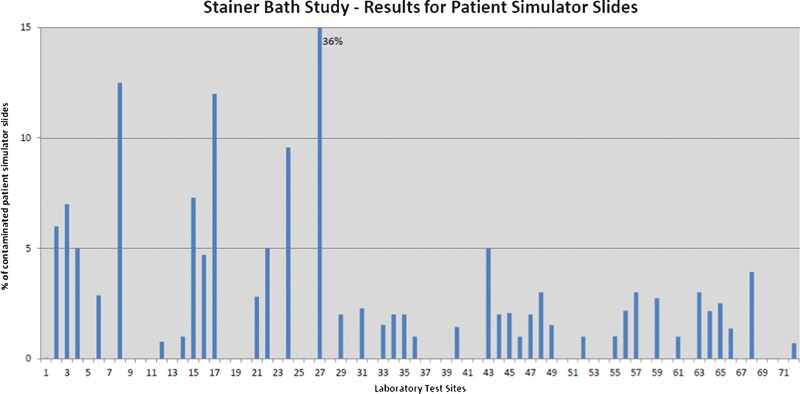
Percentage of patient simulator slides exhibiting migration of extraneous tissue or ‘floaters’ from the linear staining baths during hematoxylin and eosin (H&E) staining.

**Figure 3:**
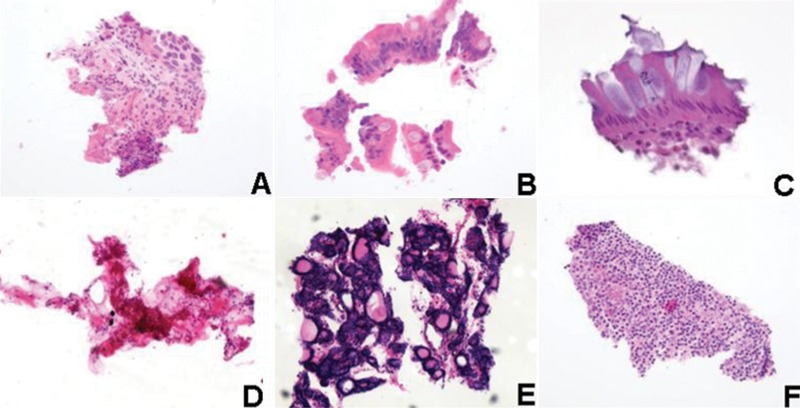
Tissue fragments and tissue types found on the patient simulator slides that may pose diagnostic challenges: (A) squamous cell carcinoma, ×200; (B) intestinal epithelium with goblet cells, ×400; (C) intestinal epithelium with goblet cells, ×400; (D) fibrous connective tissue, ×50; (E) atypical thyroid epithelium, ×50; (F) lymphoid, ×200.

Patient simulator slides were evaluated for the presence of one or more qualified tissue contaminants which migrated from one of the stainer baths in the laboratory’s linear stainer onto the blank patient simulator slides. The percentage of contaminated patient simulator slides for each laboratory is shown in Fig. 2. Fifty-seven percent (57%) of laboratories exhibited evidence of extraneous tissue migration from the stainer baths to the blank slides intended to simulate patient slides. Photographs of the tissue fragments found on the patient simulator slides are shown in Fig. 3A–F.

**Figure 4:**
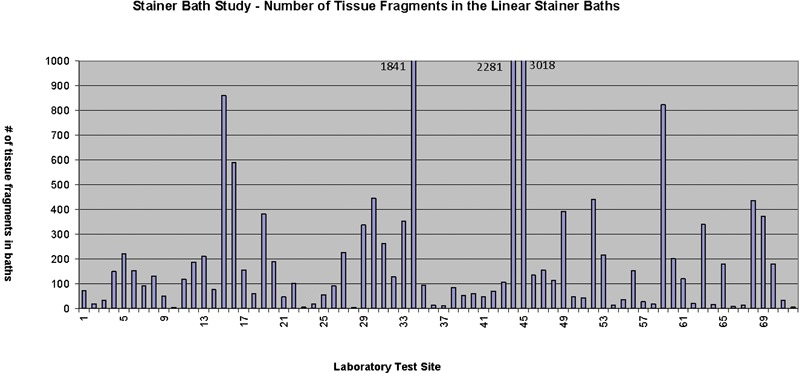
Number of extraneous tissue or ‘floaters’ present on the CytoSpin slides, reviewed by a third-party pathologist.

The total numbers of qualified tissue floaters identified on CytoSpin slides prepared from the selected linear stainer bath contents are highlighted in Fig. 4. The amount of qualified contaminants spanned from 3 to 3018, with no contaminant-free stainer baths at participating laboratories.

**Figure 5:**
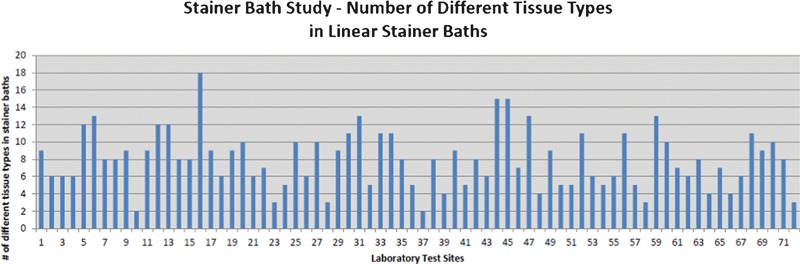
The number of different tissue types represented in linear stainer baths from 72 stainers.

**Figure 6:**
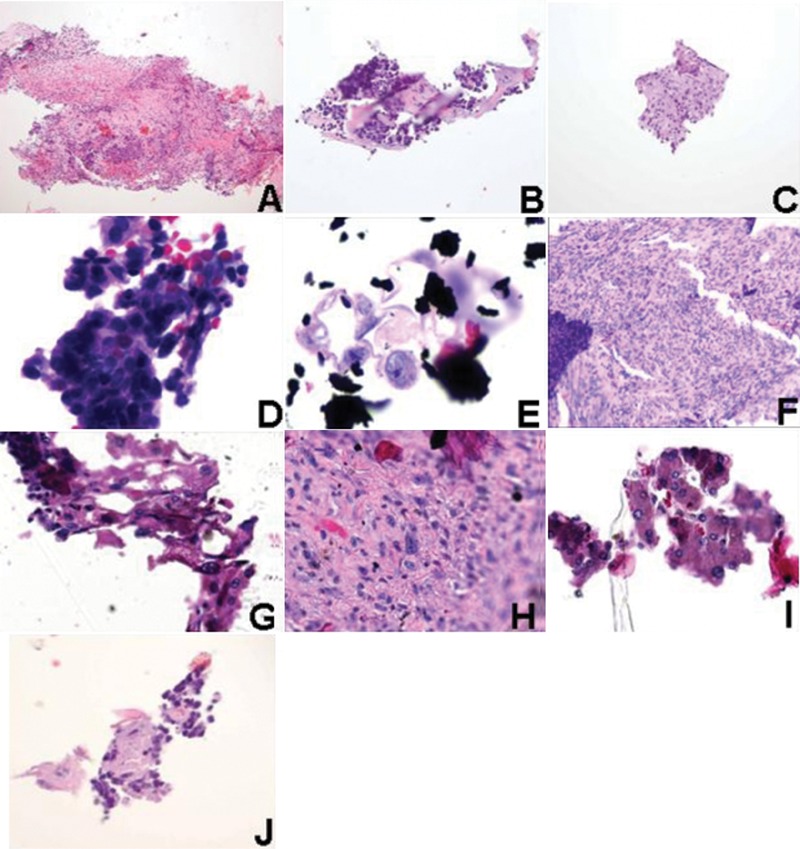
Examples of tissue fragments found in the linear stainer baths: (A) 95% alcohol bath, invasive poorly differentiated carcinoma, ×100; (B) 95% alcohol bath, invasive poorly differentiated carcinoma, ×200; (C) clearing agent bath, squamous cell carcinoma, ×200; (D) clearing agent bath, atypical squamous epithelium, ×400; (E) clearing agent bath, malignant neoplasm, NOS, ×400; (F) 100% alcohol bath, fibrous sarcoma, ×50; (G) 100% alcohol bath, atypical squamous epithelium, ×200, (H) clearing agent bath, sarcoma, ×200; (I) 95% alcohol bath, oncocytic epithelium, ×200; (J) 100% alcohol bath, poorly differentiated carcinoma, ×400.

The number of different tissue types found in the linear stainer bath contents is shown in Fig. 5. The third-party pathologist characterized a variety of different tissue types, including bloody or friable tissue types, like those shown in Fig. 6A–J.

CytoSpin slides created from the contents of the linear stainer baths were analyzed for extraneous tissue contamination. The CytoSpin slides exhibited the amount of tissue loss from patient slides into the stainer baths. Photographs of representative extraneous tissue fragments found on the CytoSpin slides are shown in Fig. 6A–J.

**Figure 7:**
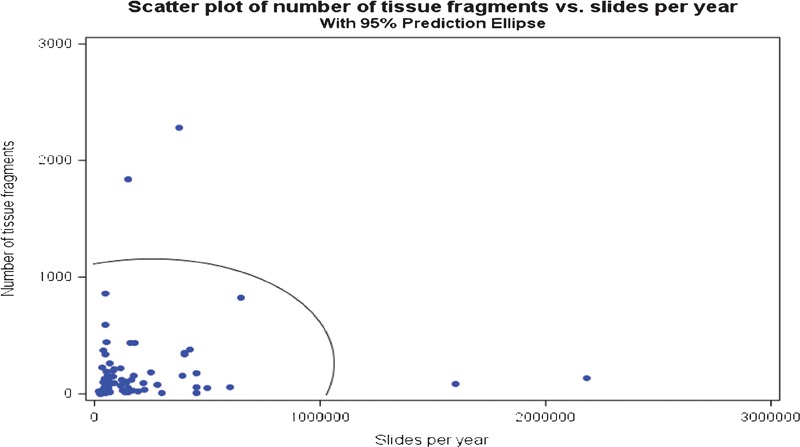
Number of tissue fragments found in the studied baths compared to the hematoxylin and eosin (H&E) annual slide volume of the participating laboratories.

**Figure 8:**
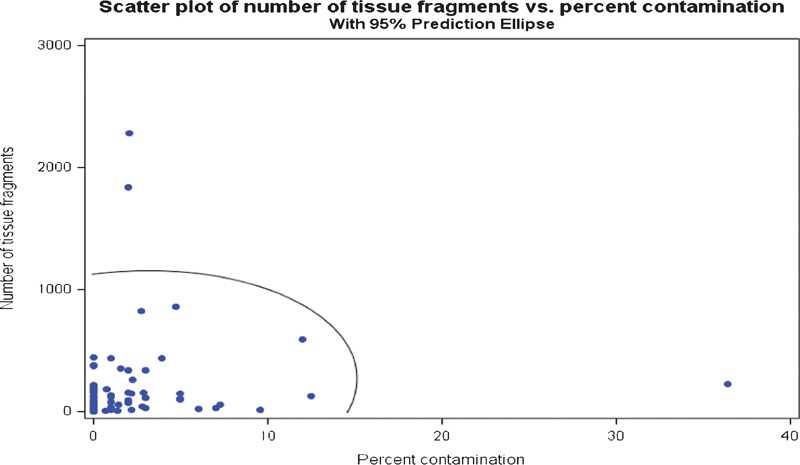
Number of tissue fragments found in the studied baths compared to the percent contamination found on the patient simulator slides.

**Figure 9:**
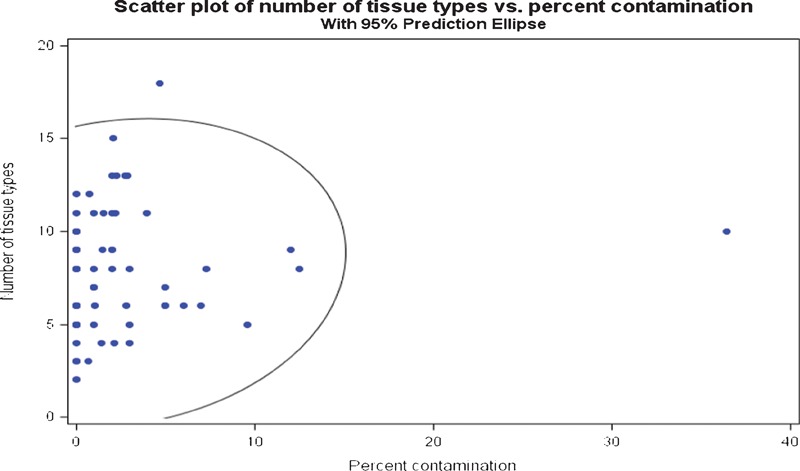
Number of tissue types found in the studied baths compared to percent contamination found on the patient simulator slides.

**Figure 10:**
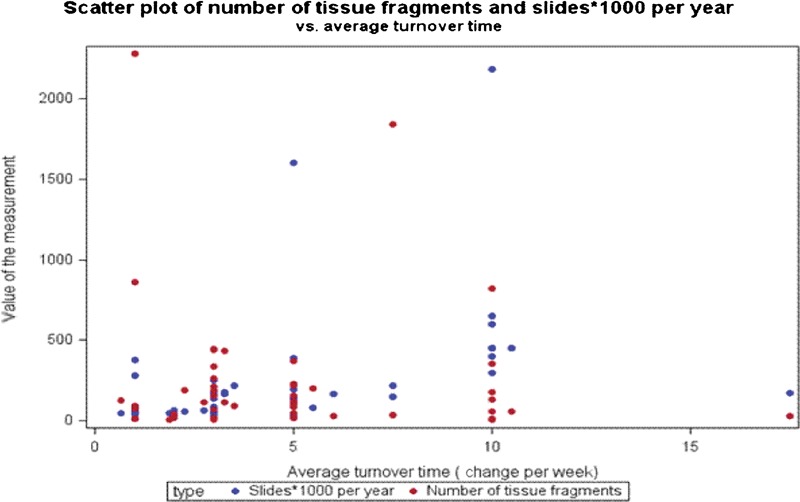
Relationship of clearing agent turnover frequency and number of tissue fragments found in the studied baths. The 5 on the x-axis indicates reagent turnover of once per day and the 10 on the x-axis indicates reagent turnover of twice per day.

Additional statistical analyses comparing floaters and slide volume, as well as reagent turnover are presented in Figs. 7–10. The scatter charts illustrate the presence of tissue fragments or cellular material during the linear staining process.

The statistical analysis demonstrated the relationships between the slide volume, reagent turnover, tissue types, and contamination rate. A direct relationship between slide volume and overall contamination rate was expected. To visualize the relationship between slide volume and overall contamination, a graphical summary with data points was presented. The data points located inside the ellipse represent the 95% confidence level of expected rate of contamination.

## Discussion

This global, multi-laboratory study showed significant loss of extraneous tissue fragments from patient slides to stainer baths and tissue migration from the stainer baths onto blank patient simulator slides. The sharing of reagents during linear H&E staining contributed to a variable amount of tissue cross-contamination among simulated patient slides. Even though the level of contamination in the stainer baths differed, every participating laboratory showed evidence of tissue contamination, e.g. benign tissue fragments, as well as atypical and malignant fragments. The number of different tissue types in the baths ranged from 2 to 18, with an average of 8 per linear stainer.

Further evaluation of preanalytical variables that could contribute to tissue discohesion is outside the scope of this study. However, future examination of preanalytical variables in the laboratory that address extraneous tissue contamination may include inadequate tissue fixation time, slide wicking technique to remove excess water after the section is picked up from the waterbath, use of positively charged slides, and slide drying method before staining. It seems unlikely that any modification to preanalytical variables in the laboratory would mitigate extraneous tissue contamination in the linear stainer baths completely.

The statistical analysis reveals that there is no correlation, pattern, or other determining factors which might influence the presence of extraneous tissue in the reagent baths (confidence interval = 95%; Figs. 7–9). Reagent turnover did not appear to mitigate the apparent randomness of the presence of extraneous tissue fragments. Further, there is no apparent relationship between laboratory slide testing volume, number of contaminants found in the stainer baths, or number of identified tissue types in the stainer baths (Figs. 7–9). The magnitude of extraneous tissue cross-contamination, in terms of number of tissue types and total number of fragments found in the stainer baths, is independent of slide volume and reagent turnover in the stainer baths (Fig. 10). That is, a small volume laboratory is just as likely to experience a high degree of tissue cross-contamination as a large volume laboratory. Similarly, how often laboratories changed or filtered reagents had no effect on the extraneous tissue contamination found in reagent baths. The study results indicate that stainer bath contamination is a random process.

In 2011, a study by Carpenter described the contamination in stainer baths and its impact on pathologist interpretation of patient samples.^5^ The time and effort required to determine the origin and the identity of a floater interrupt the workflow in the histology laboratory. Additional sectioning and staining, as well as reading time for the pathologist, slow the result reporting to the clinician, and may delay treatment for the patient.

Carpenter and Gephardt agree that a floater can cause a diagnostic dilemma.^4^^,^^5^ Carpenter made the following points about floaters. For example, adenocarcinomas of various organs strongly resemble one another and are, therefore, particularly problematic floaters. Not having the ability to determine the origin of extraneous tissue poses challenges for the reviewing pathologist. For pathologists specializing in and reading similar tissue slides frequently throughout the day, contamination poses an increased risk of causing misidentification among patient specimens. Patient tissue stands a higher likelihood of encountering a tissue contaminant with similar characteristics. Floaters with similar morphologic structure can certainly cause confusion between the native tissue on the slide and tissue that is from another patient sample. The occurrence of ovarian tissue on a prostate biopsy slide would more easily be recognized as a floater versus an incident in which a malignant ovarian fragment transferred to another ovarian tissue slide.

For cases in which microscopy alone cannot distinguish contamination from patient tissue, Shibata suggests that the use of molecular identity testing or even re-biopsy may be needed.^6^ DNA identification testing is time consuming and of substantial cost, while re-biopsy exposes the patient to additional procedure-related risk and is not always possible.^5^ If DNA analysis is required outside the anatomic pathology laboratory to determine the patient’s identity, the time to present a result to the clinician could be extended.^6^ In addition, the credibility and reputation of the laboratory are at risk, if only from the reporting delay needed to fully evaluate a case with slide contamination. Our study demonstrated that mitigation of tissue cross-contamination during linear staining can be a challenge and the histopathology laboratory is not alone in realizing the risk. The College of American Pathologists (CAP) identified mitigation of cross-contamination as a requirement on the CAP checklist for the cytology laboratory and a recent editorial by Fischer supported the importance of minimizing risk for cross-contamination in cytology samples.9 The CAP checklist does not include a mitigation requirement for histopathology, but our data suggest that the risk is similar to cytology laboratories, though mitigation strategies using filtering and changing of reagents may be insufficient based on the data from this study.

## Conclusion

The study results determined that extraneous tissue contamination exists within the linear staining process and, despite best efforts, traditional practices such as filtering and changing reagents are unsuccessful in reducing tissue contamination. Contamination risks are an obvious threat to the laboratory from a reputation and financial perspective, but the associated productivity issues for the technologists and pathologists may justify considering a change to current practice. Additional scrutiny in identifying and categorizing extraneous tissue may lead to longer review times and reduced productivity. Because of both the threat to patient identification and laboratory productivity, opportunities for further investigation of extraneous tissue contamination should be considered. Subsequent research might focus on whether individual slide staining would more effectively decrease the potential for cross-contamination, what other strategies might be employed to reduce the chance for extraneous tissue, and what effect extraneous tissue identification has on patient outcomes and laboratory productivity. Laboratories using linear stainers should consider adopting a rigorous documentation and investigation procedure to use when tissue floaters are identified by the pathologist.
